# Pterygospinous Bar and Foramen in the Adult Human Skulls of North India: Its Incidence and Clinical Relevance

**DOI:** 10.1155/2014/286794

**Published:** 2014-05-20

**Authors:** Anjoo Yadav, Vinod Kumar, Richa Niranjan

**Affiliations:** ^1^Department of Anatomy, Government Medical College, Kannauj 209732, India; ^2^Department of Anatomy, Government Medical College, Orai, India; ^3^Department of Anatomy, Government Medical College, Haldwani, India

## Abstract

Study of skulls has attracted the attention of anatomists since ages and sporadic attempts have been made to study skulls from time to time. Talking about the pterygoid processes of sphenoid bone, the irregular posterior border of lateral pterygoid plate usually presents, towards its upper part, a pterygospinous process, from which the pterygospinous ligament extends backwards and laterally to the spine of sphenoid. This ligament sometimes gets ossified as pterygospinous bar and a foramen is then formed, named pterygospinous foramen, for the passage of muscular branches of mandibular nerve. The present study was undertaken to observe the incidence and status of pterygospinous bony bridge and foramen, its variations, and clinical relevance in the adult human skulls of North India. For this purpose, 500 skulls were observed, belonging to the Anthropology Museum of Department of Anatomy, GSVM Medical College, Kanpur. Pterygospinous bars were found to be present in 51 skulls (10.2%), out of which completely ossified pterygospinous bony bridges were present in 20 skulls (4%) while 31 skulls (6.2%) had incompletely ossified pterygospinous ligaments. Such variations are of clinical significance for radiologists, neurologists, maxillofacial and dental surgeons, and anaesthetists, too.

## 1. Introduction

The vertebrate skull is the most modified part of axial skeleton and its study has attracted the attention of anatomists and anthropologists since ages. Sporadic attempts have been made to study skulls, from time to time, in the past centuries but its individual bones and their ligaments in particular have somehow not received as much attention.

Sphenoid bone lies in the base of skull, “wedged” between the frontal, temporal, and occipital bones. It has a central body, paired greater and lesser wings spreading laterally from it, and two pterygoid processes. Each of these processes, descending perpendicularly from the junctions of greater wings and body of sphenoid, consists of a medial and a lateral plate. The medial pterygoid plate is narrower and longer while the lateral pterygoid plate is broad, thin, and everted; a variable pterygospinous process on its irregular posterior border is connected by a ligament (sometimes ossified) to the sphenoid spine [1].

Jones [2] states that lateral pterygoid plate is broader and shorter than the medial and is directed backwards and slightly laterally. Its posterior border usually presents, towards its upper part, a sharp spine from which the pterygospinous ligament extends backwards and laterally to the spine of sphenoid. This ligament sometimes gets ossified and a foramen is then formed, named pterygospinous foramen, for the passage of muscular branches of mandibular nerve. Sometimes there is another spine towards the lower end of this border for another pterygospinous ligament.

Breathnach [3] states that a projection, more or less prominent on the posterior edge of the lateral pterygoid plate, a little way down, may mark the anterior attachment of a “pterygospinous” ligament extending to the base of the sphenoidal spine. The ligament may be short, fastened higher up on the plate or two ligaments, long and short, may be present: ossification may extend someway into these, so that a bony bar may be present here. The nerves issuing from the foramen ovale have varying relations with these bands, which are probably modified fibres of the lateral pterygoid muscle.

## 2. Aims

Ossified pterygospinous ligament seems to be a major cause of entrapment of lingual nerve or a branch of mandibular nerve and may cause mandibular neuralgia. Therefore, this present study was undertaken to determine the incidence of pterygospinous bony bridge and foramen and its variations in the adult human skulls of North India, to discuss its clinical relevance.

## 3. Material and Method

For the present study, a total of 500 skulls were observed, randomly selected from the stock of about 1300 skulls, belonging to the Anthropology Museum of Department of Anatomy, GSVM Medical College Kanpur. The age and sex of the macerated skulls were not taken into consideration. The skulls were washed and their bases were closely observed in regards with the pterygoid plates of sphenoid, for the presence of ossified pterygospinous bars and foramina. The various measurements like PS bar length and breadth and PS foramen diameter were measured with the help of vernier calipers.

## 4. Observations

Osseous bars were either complete or incomplete, that is, if bony bridges were extending from lateral pterygoid plate to sphenoid spine apex, it was termed complete, and if pterygospinous ligament failed to make a contact with sphenoid spine, it was considered incomplete. In case of presence of complete pterygospinous bar, a well-formed pterygospinous foramen was present while, in case of incomplete bar, partial foramen was formed.

**Figure 1:**
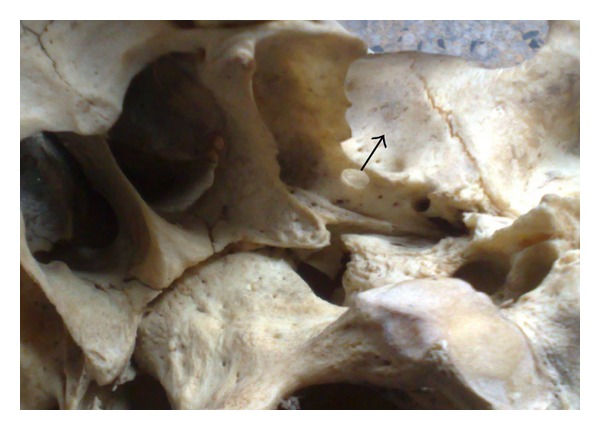
Completely ossified pterygospinous bar and foramen on the right side (arrow).

**Figure 2:**
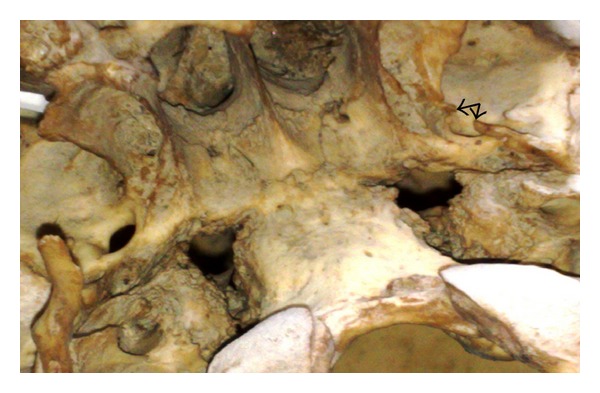
Incompletely ossified pterygospinous ligament on the right side (arrows).

**Figure 3:**
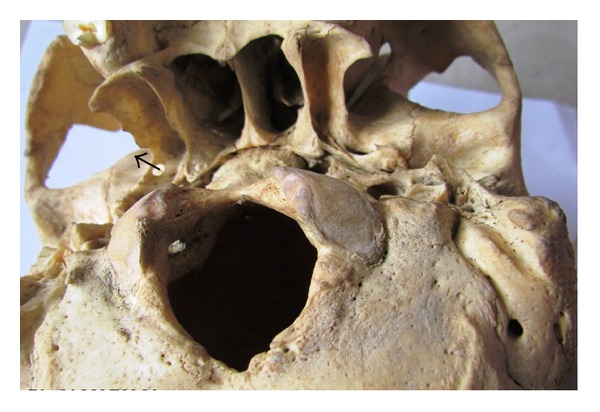
Complete pterygospinous bar and foramen on the left side (arrow).

**Figure 4:**
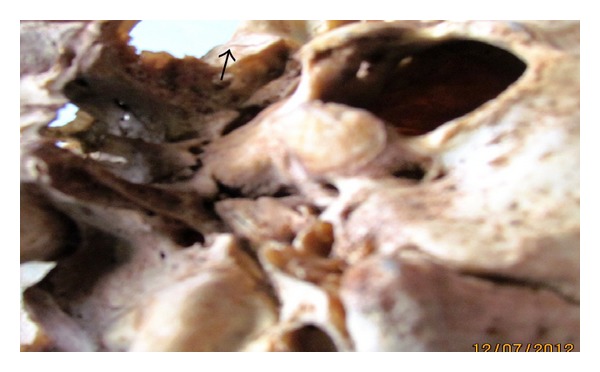
Completely ossified pterygospinous bar on the right side (arrow).

Pterygospinous bars were found to be present in 51 skulls (10.2%), out of which completely ossified pterygospinous bony bridges (as in Figure 1) were present in 20 skulls (4%) and in none of the cases it existed bilaterally. It was present in 14 skulls on the right side (as in Figure 4) and in 6 skulls on the left side (as in Figure 3)—a total of 31 skulls had incompletely ossified pterygospinous ligaments (as in Figure 2)—in 25 skulls, it was present unilaterally (in 13 skulls on the right and in 12 skulls on the left), and 6 skulls had it bilaterally. All these findings have been recorded in Table 1.

As far as pterygospinous bony bar measurements are concerned, in case of complete PS bridging, length of the PS bar ranged from a minimum of 5 mm to a maximum of 10.5 mm while breadth of the PS bar varied from 1.5 mm to 3 mm; the maximum diameter of the PS foramen ranged from 5 mm to 10.5 mm.

In case of incomplete bridging, the gap or distance between the two nonmeeting ends ranged from 3 mm to 7 mm. All these distances had been recorded on either side and their mean values have been presented in Table 2.

## 5. Discussion

Several ligaments are present in relation to sphenoid bone at the base of skull such as pterygospinous, interclinoid, caroticoclinoid, and pterygoalar ligaments. Ossification of these ligaments may have different clinical implications. The pterygospinous ligament, described by Civinini in 1835 (cited by Tebo [4]), is directed from the spine of sphenoid to the pterygospinous process, when ossified, establishing the pterygospinous foramen, also known as Civinini foramen. The incidence of pterygospinous bony bridges has been reported by different authors with different results, as can be seen in Table 3.

Wood [5] reported an 8% pterygospinous ligament ossification in Hawaiian skulls.

Krmpotić-Nemanić et al. [6] reported ossified pterygospinous ligaments in 5 out of 100 skulls and emphasized that these bony bridges may be one of the reasons of mandibular neuralgia.

Kapur et al. [7] reported a prevalence of 18.36% of pterygospinous bar in a sample of 305 Croat skulls. Complete ossification of pterygospinous ligament was found in 3.6% skulls—1.31% bilaterally as well as 1.31% on the right side and 0.98% on the left side. Incomplete type was found in 14.7% skulls—bilaterally in 12 skulls and unilaterally in 33 skulls. They emphasized that the presence of such an ossified PS ligament may prevent anaesthesia of mandibular nerve at the lateral subzygomatic approach.

Peker et al. [8] studied 452 adult dry crania in Anatolian population and observed completely ossified pterygospinous ligament in 5.5% skulls. In 14 out of 452 skulls (3.1%) complete pterygospinous osseous bridges were bilateral. The frequency of complete pterygospinous bony bridges was 4.2% on the right side and 6.4% on the left. The course of the branches of mandibular nerve was apparently affected by the ligament.

Atamaz-Pinar et al. [9] found completely ossified pterygospinous ligament in 12 cases out of 361 dry adult human crania and incompletely ossified ligaments in 35 cases.

Lüdinghausen et al. [10] reported that a complete osseous bar, arch, or lamina connecting the posterior border of lateral lamina of pterygoid process and sphenoidal spine existed in 6 of the 100 human dry skulls and 1.85% in cadavers.

Nayak et al. [11] examined 416 dry human skulls of Indian (Dravidian) origin for pterygospinous bony bar and found total incidence to be 9.61%, incomplete pterygospinous foramen in 3.84%, and complete pterygospinous bar in 5.76% skulls.

Antonopoulou et al. [12] observed 50 Greek dry skulls and reported completely ossified pterygospinous bridge in only 1 skull bilaterally and incomplete ossification in 25 out of 100 observations. These observations were made out in a three-dimensional reconstruction in a CT image.

Shinde et al. [13] studied a total of 65 skulls and only in 2 cases found incompletely ossified pterygospinous ligament—in one case it belonged to the left side and in the other it belonged to the right. There was a small gap between the spine of sphenoid and the posterior border of lateral pterygoid plate which measured 3 mm in both cases.

Agarwal et al. [14] studied 67 adult human skulls of Punjab region and revealed the incidence of pterygospinous bar as 9.7%—complete pterygospinous bridges in 2.99% and incomplete ones in 6.72% of skulls.

Verma et al. [15] carried out their study on 116 macerated adult human skulls and reported a total incidence of 18.1%.

The present study reports an incidence of 10.2%—which is in sync with the earlier studies of Indian skulls by Nayak et al. [11] and Agarwal et al. [14].

Complete ossification of pterygospinous bar, resulting in a well-formed pterygospinous foramen, was reported in 20 out of 500 skulls studied in the present study while few authors like Das and Paul [16] found only incompletely ossified pterygospinous ligament—only 1 case in 50 skulls. Similarly, Shinde et al. [13] observed only incompletely ossified PS ligament in 2 out of 65 skulls.

Bilateral presence of complete PS bars/bridges has not been reported either by us or in the study by Verma et al. [15].

In most of the above studies, the incomplete variety was more common than the complete one.

These osseous variations are important not only in anatomy but also in clinical practice [12]. The distribution pattern of the mandibular nerve was affected by the positioning of the pterygospinous bar and ligament. It is likely that in humans this bony bar represents a phylogenetic remnant.

The presence of complete or incomplete pterygospinous bar is related with some important structures present in this region like mandibular nerve, as it comes out of foramen ovale, and its branches, otic ganglion, middle meningeal artery and vein, tympanic nerve, and medial and lateral pterygoid muscles. These structures may get compressed against these bony formations and can produce many clinical symptoms like pain, especially during chewing, and could provoke trigeminal neuralgia [6].

Peuker et al. [17] were the first to demonstrate the presence of ossified pterygospinous ligament causing compression of lingual nerve between the bony bridge and medial pterygoid muscle, which results in lingual numbness and pain, associated with speech impairment.

Considering the close relationship of the chorda tympani nerve, it may also be compressed by the anomalous bar of bone and its involvement would result in abnormal taste sensation in the anterior two-thirds of the tongue [16]. Such compression may also produce a partial lesion of the nerve, which can lead to a distortion of signaling patterns or ectopic impulses within the damaged nerve fibers.

In the presence of an ossified pterygospinous ligament, the main trunk of mandibular nerve is redirected laterally and its dividing neural routes (lingual nerve and inf. alveolar nerve) have to cross the extended lateral pterygoid plate. Because of this abnormal course, there is greater risk for neuralgia occurring due to the nerves becoming entrapped or compressed between the osseous structures and muscles [10]. A wide pterygospinous bar exists in all skulls of herbivores, rodentia, carnivores, and mature monkeys.

The pterygospinous bony bridge can also pass among the fibers of the lingual nerve and divide it into anterior and posterior parts. Anterior part passes medially and lies between tensor veli palatini muscle and the bony bridge, so these fibers are vulnerable to the risk of compression [18].

Causes of ossification of pterygospinous ligament may not be known but these formations were more often present in males and unilateral presence was more common than bilateral one. While applying conductive anaesthesia on the mandibular nerve by lateral subzygomatic route, presence of these ossified structures at the lateral plate's posterior border of pterygoid process should be well considered and verified [7].

Lateral pterygoid plate forms an important landmark for mandibular anaesthesia and any anomaly in the lateral pterygoid plate is bound to confuse anaesthetists.

Considering the phylogenetic and clinical significance of these pterygospinous bars and foramina, the present study is very important especially for surgeons, anaesthetists, dentists, anatomists, and anthropologists to know the types of osseous bridges and their incidence in this region of cranial base.

There may be failure of anaesthesia in cases of treatment of trigeminal neuralgia due to the presence of an ossified pterygospinous ligament, or it can also constitute an obstacle for the mandibular nerve block that is a preferred method for pain relief specially in fractures of mandible or cancer patients [9, 17].

## 6. Conclusion 

Out of 500 studied skulls, the presence of pterygospinous bars and broad lateral pterygoid plates was reported in 51 (10.2%) skulls—completely ossified pterygospinous ligament in 20 skulls (4%) and incomplete ossification of pterygospinous ligament in 31 skulls (6.2%). Different authors have reported different results, though, and this difference is probably regional; that is, in every study the skulls observed belonged to different topographical or geographical regions.

Knowledge of these anatomical variations is important because ossification of pterygospinous ligament can result in formation of a foramen through which mandibular nerve branches may pass in most of the cases which may get compressed, depicting various clinical symptoms, depending upon the dimensions of the pterygospinous foramina and grades of compression.

Therefore, this study is important to radiologists and neurosurgeons, maxillofacial and dental surgeons, and anaesthetists along with anatomists and anthropologists.

## Figures and Tables

**Table 1:** Incidence of pterygospinous bar and foramen in present study.

Total skulls observed	500			
(1) Pterygospinous bar present in	51 (10.2%)			

(2) Incompletely ossified in	31 (6.2%)	U/L	Rt	13
			Lt	12
		B/L	06	

(3) Completely ossified in	20 (4%)	U/L	Rt	14
			Lt	06
		B/L	00	

**Table 2:** Various measurements regarding PS bridging.

(1) In case of complete pterygospinous bridging	Mean length of PS bar	Rt side	Lt side
		7.8 mm	8.0 mm
	Mean breadth of PS bar	2.0 mm	1.8 mm
	Max. diameter of PS foramina	7.8 mm	8.0 mm

(2) In case of incompletely ossified pterygospinous bar	Gap/distance between the 2 separate ends	5.0 mm	5.0 mm

**Table 3:** Comparative table for incidence of ossified pterygospinous ligament.

S. number	Research worker	Year	Skulls studied	Total incidence	Incompletely ossified PS ligament in	Completely ossified PS bar in
1	Wood [5]	1931	Hawaiian	8%		
2	Kapur et al. [7]	2000	Croats	18.36%	14.7%	3.6%
3	Peker et al. [8]	2002	Anatolian	5.5%	—	5.5%
4	Lüdinghausen et al. [10]	2006	German	6%	—	6%
5	Nayak et al. [11]	2007	Indian	9.61%	3.84%	5.76%
6	Antonopoulou et al. [12]	2008	Greek	14%	12%	2%
7	Shinde et al. [13]	2011	Indian	3.07%	3.07%	—
8	Agarwal et al. [14]	2012	Indian	9.7%	6.72%	2.98%
**9**	**Present study**	**2013**	**Indian**	**10.2%**	**6.2%**	**4%**
